# Text Mining and Quantitative Research of Medical Service Policy: Sichuan Province as an Example

**DOI:** 10.3389/fpubh.2020.509842

**Published:** 2021-01-08

**Authors:** Huiqin Zhang, Liping Zhu, Chen Zeng, Xudong Chen

**Affiliations:** College of Management Science, Chengdu University of Technology, Chengdu, China

**Keywords:** medical service policy, text mining, policy instruments, policy strength, medical service activities

## Abstract

Medical service policy plays a prominent role in the development of a “Healthy China.” This paper constructs a three-dimensional framework for text mining of medical service policy using the elements of policy instruments, policy strength, and types of medical service activity. Taking Sichuan Province as an example, 221 medical service policy documents, issued by the government and related departments, are selected as the research sample; the policy instruments, policy strength, and medical service activity types are analyzed using ROST and Nvivo 11.0 software. It is concluded that the government needs to optimize the structure of policy instruments, to appropriately reduce the use of environmental policy instruments in particular, while increasing the use of demand-based policy instruments. It is necessary to strengthen the interaction between the various sub-policy instruments, and to increase the use of financial services, fiscal taxes, overseas communications, and strategic measures. An increase in the implementation of government policy assists the acceleration of the policy landing, the further improvement of the supervision system, and the safeguard mechanism of the three medicine policy linkage, which can improve the sustainability of the medical service policy, and further resolve the difficulty and expense of seeing a doctor.

## Introduction

Health is the foundation of a better life for everyone, and, increasingly, attention has been paid to factors affecting health, ranging from global environmental problems (1, 2), climate change (3, 4), public health issues (5, 6), the impact of urbanization (7), medical health provisions (8, 9) the level of medical care in a country or region (10), disease transmission (11), health care (12, 13), quality of life (14), and lifestyle (15, 16). One of the most important factors in ensuring the health of the population is a focus on medical problems (17, 18). The realization of “national health” requires China to provide universal health security for all citizens (19), especially in terms of medical and health policy. The Healthy China 2030 Planning Outline (20) states that the promotion of a Healthy China requires the development of appropriate legal frameworks in the field of healthcare, to create a comprehensive health service system. China is committed to building such an integrated medical and health service system, with a clear division of labor, complementary functions, close cooperation, and efficient operation by 2030. Policies containing authoritative guidance can define the objectives of the challenge and the measures required in accordance with the prescribed principles of action to carry out clear tasks in an orderly manner; the research into, and subsequent formulation of, medical service policy fundamentally affects the effectiveness of its implementation, which determines the quality of medical services to a great extent.

Advice on progressing the reform of the medical and health system in 2019, which was issued by the General Office of the State Council, clearly identified the need to study and formulate relevant policy documents; guidance, management, assessment methods; and regulations on medical and health care within 1 year, in order to implement the Healthy China strategy and further reform the medical and health system. Therefore, in-depth analysis of the content of the medical service policy document is of great practical significance to the improvement of the medical service and the promotion of the “health of all people.” At present, there are many problems to be solved in China's medical service (21). The assumption that “Difficulties and high expense in medical care” has been the general attitude toward health care in general; furthermore, the regional distribution of medical and health care resources is not equal (22). In addition, medical reform measures are still in the initial scoping stages. There is no stable and sound system (23) and the reform experience is not mature (24). Sichuan, a province in the west of China with a large population, has a broad geographical area, and a complicated and poorly developed medical service. This paper scrutinizes the medical service policy document of the province, issued after the implementation of comprehensive medical reform, and uses the text mining function of ROST and Nvivo 11.0 software to perform quantitative analysis of the policy according to the following three dimensions: policy instruments, policy strength, and medical service activity types.

## Literature Review

### Policy Instruments

Policy instruments (25), which are also known as “government tools” or “governance tools,” have been used effectively in many industries to resolve development problems (26–28). Medical policy instruments are a series of means, technology, methods, and mechanisms to realize the policy goal of effective and fair supply of medical services. There are a number of classifications of policy instruments in academia (29). Rothwell and Zegveld (30) divide policy instruments into three types, namely supply, environment, and demand; Huang et al. (31) undertook a text analysis of policy instruments adopted by the central government of China on the basis of this classification. Wang (32) used Rothwell and Zegvelad's policy instrument classification in their study of China's elderly service industry and used it as the X dimension of the analysis of the policy related to this. Linder and Peters (33) proposed that the government tools are multicomponent, and include order clauses, financial aid, control regulations, taxation, exhortations, authority, contracts, and so on. Howlett and Ramesh (34) divided policy instruments into voluntary tools, hybrid tools, and mandatory tools based on the level of government intervention. According to the degree of government enforcement and functional differences, and on the basis of classifications by Howlett and Ramesh (34) and other scholars, Wang (35) concluded that there are four types of policy instrument: mandatory, market, guiding, and voluntary. Xiong (36) divided policy instruments into 11 secondary analysis categories under the category of health policy instruments, e.g., family and community, voluntary organizational, and market, again based on the classification provided by Howlett and Ramesh (34). Chen (37) categorized government tools into imperative tools, incentive tools, capacity building tools, and system change tools.

**Figure 1:**
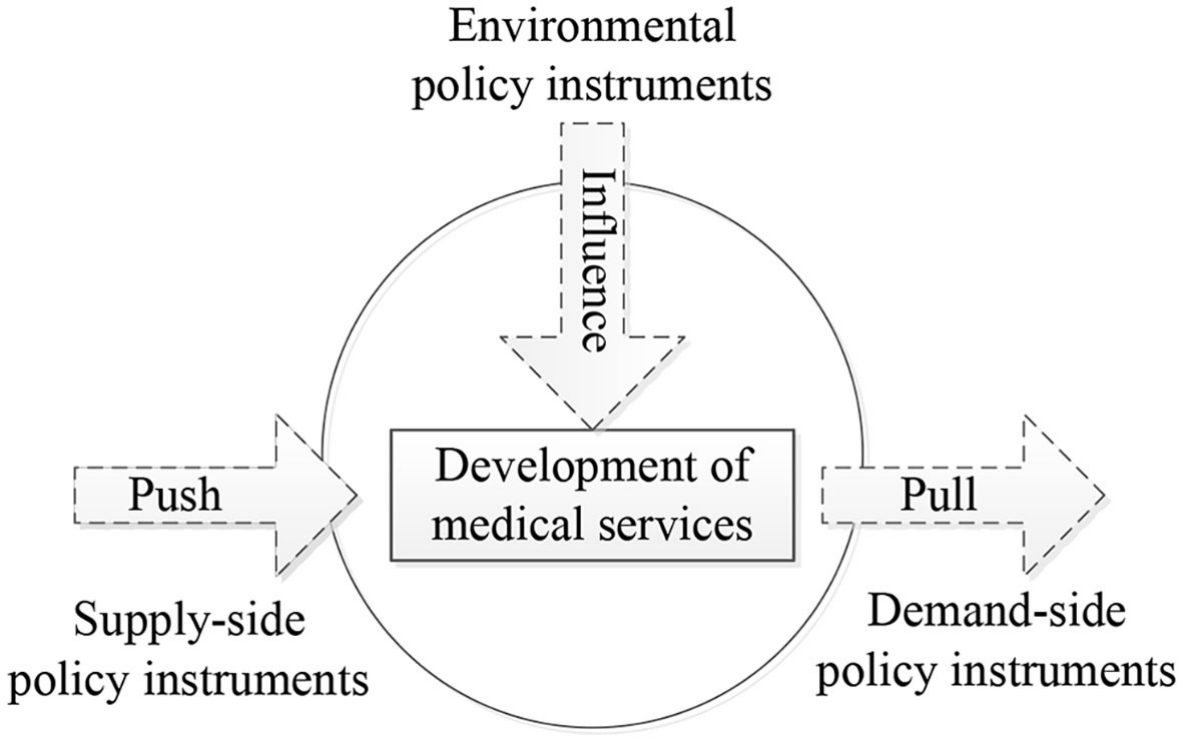
The policy instrument type dimension of medical policy text analysis.

Rothwell and Zegveld's policy instrument classification gave relatively little importance to plays down their mandatory characteristics and strengthens the role of government as environmental architect in the process of promoting policy projects, rather than only as an interventionist and controller (32), which is consistent with the basic principles of medical policy development. Secondly, in terms of feasibility, the sub-policy instruments of this classification method are more specific, and the operation methods are clearer. Considering their applicability in theory and their maneuverability in reality, this paper will make some adjustments to the sub-categories according to the classification of Rothwell and Zegveld. The policy instrument type dimension of medical policy text analysis is constructed as the X dimension, which is shown in Figure 1. The supply-side policy instruments refer to the government's direct expansion of the supply of medical services through providing support in the form of personnel, information, technology, funds, and so on, in order to improve the supply of related elements of medical development and promote the improvement of the overall service level of medical services. Environmental policy instruments enable the government to influence the environmental factors affecting medical development through policies involving finance, the tax system, and regulation, and to provide a favorable policy environment for medical activities, to indirectly influence and promote the improvement of the medical and healthcare service level. The demand-side policy instruments actively open up and stabilize the medical service market, which is a driving force for market demand.

### Policy Strength

The strength of a policy depends on the administrative level of the relevant government agencies: the higher the administrative level of a government agency, the greater its authority. This phenomenon reflects the government's priority on medical development. The weaker policy was promulgated by the lower-level government agencies, which also reflected the importance attached by the lower-level government to the development of medical care. At present, research on policy strength mainly focuses on quantitative research. Cheng and Qian (38) cited Peng et al. (39) to determine the policy strength assignment standard of “1~5 points,” in order to analyze China's technological innovation policy. Weng et al. (40) adopted the scoring standard of “1~6 points” to quantitatively analyze the policy intensity of China's Light-Emitting Diode(LED) industrial policy, using the concept of policy quantification. Huang et al. (41) also adopted Peng Jisheng's “1~5 points” policy strength assignment standard and established a Contents-Outlook-Power-Authorities(COPA)policy analysis framework for text mining, social network analysis, and vector autoregression. In order to evaluate the policy strength more effectively, this paper adopts the standard of Peng et al. (39) to assign policy strength on a scale of “1~6 points.” In this paper, the policy strength is regarded as the Y dimension of medical policy analysis, The quantitative standards for medical service policy is shown in Table 1.

**Table 1:** The quantitative standards for medical service policy.

**Scoring standard**	**Score**
State enacted relevant laws and regulations	6
Sichuan Provincial People's Congress and its Standing Committee enacted local regulations.	5
Sichuan Provincial Government enacted regulations, etc.	4
Sichuan Provincial Government promulgated the Provisional Regulations, programs, decisions, opinions, methods, standards, etc.	3
Sichuan Provincial Government under the jurisdiction of commissions, bureaus, offices, and other views.	2
Notice, announcement, etc.	1

### Medical Service Activities

Most existing policy content analysis frameworks select two or more of the dimensions of policy instruments, policy subjects, policy strength, and so on. However, medical service policy is characterized by multi-type activities, therefore this feature is introduced here into the policy analysis framework. The types of activity related to medical service can generally be divided into three categories: medicine-related activities, medical activities, and medical insurance activities (36). In this paper, the type of medical service activity is used as the Z dimension of medical service policy analysis.

**Figure 2:**
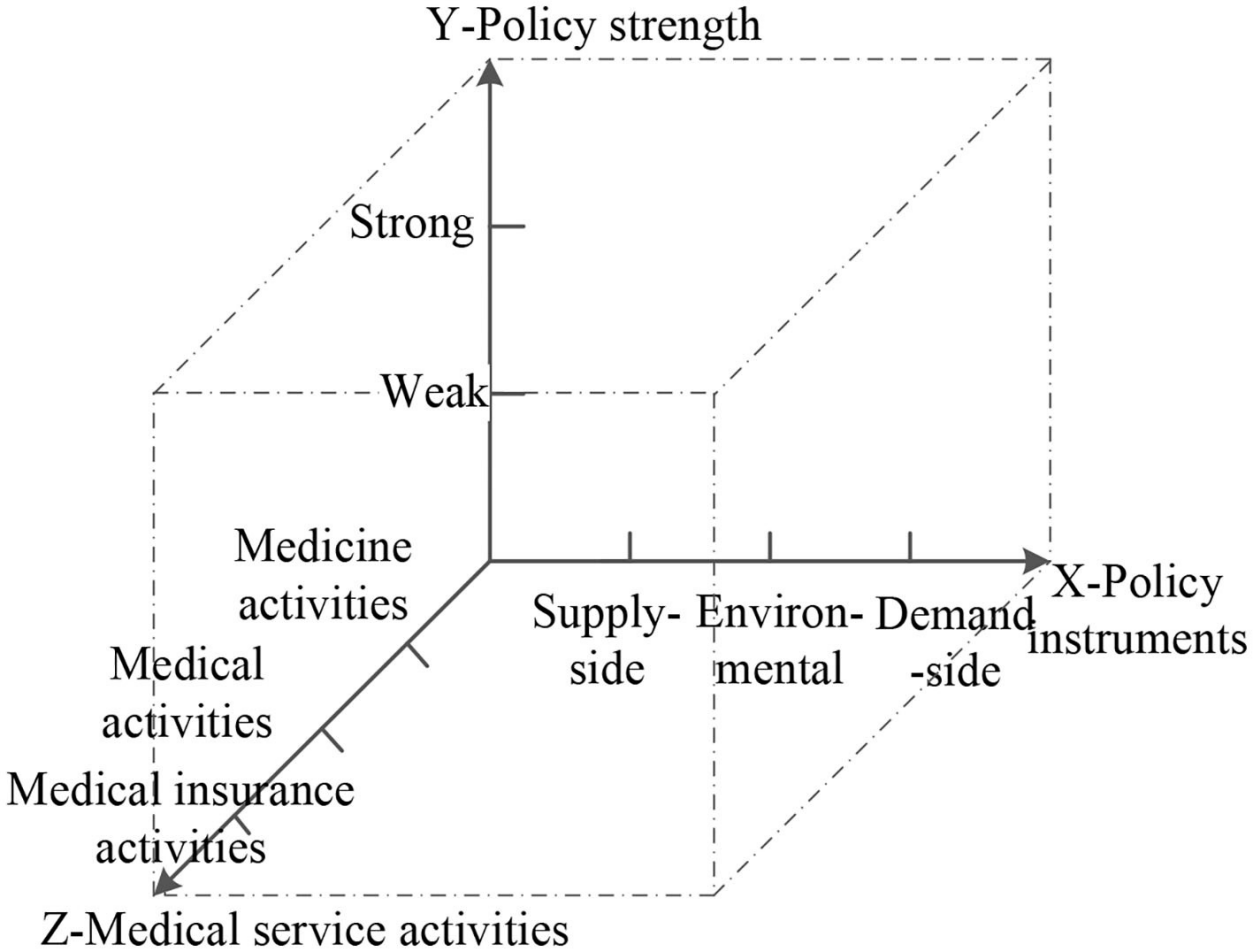
Three-dimensional analysis of medical service policy.

This paper constructs a three-dimensional framework for analyzing medical policy based on the elements of policy instruments, policy strength, and medical service activities, as shown in Figure 2; this enables interpretation of the content of medical service policy and provides a reference for use in perfecting the medical service policy system.

## Methods

### Research Methods

The research method adopted is content analysis, which enables systematic and objective analysis of the policy text, both quantitatively and qualitatively (42). This has developed into a more mature policy text analysis method by making objective rather than direct observations (43). Many scholars in China and other countries use content analysis to analyze policies.

Roumell and Salajan (44) conducted an empirical analysis of the four National Education Technology Program (NETP) documents issued by the Ministry of Education of the United States, and analyzed the evolution of its e-learning policy. Guo and Xu (45) used the content analysis method to carry out quantitative analysis of the policy text of entrepreneurship in Beijing, Shanghai and Shenzhen. Geng (46) adopted the content analysis method to study the combined policy of medical care in China from the perspective of the policy tool. Jiang and Yang (47) analyzed the evolution of the national and three regional industrial policies, and its links to the industry life cycle by using content analysis according to the three dimensions of policy strength, policy measures, and policy objectives. Alla et al. (48) applied the same method to the field of mental health policy development. Kristianssen et al. (49) identified policy decisions, issues, objectives, and measures by content analysis of the Swedish “zero vision” policy. Liu et al. (50) used the method to construct a policy analysis framework for regional innovation development from the five dimensions of time, policy subject, regional spatial level, policy content, and policy object by using the content analysis method. It is evident therefore that the content analysis method in the context of policy is well-established. This study adopts the content analysis method to analyze the collected medical policy text, and uses mainly Nvivo 11 to encode and analyze the policy text.

### Data Collection

**Figure 3:**
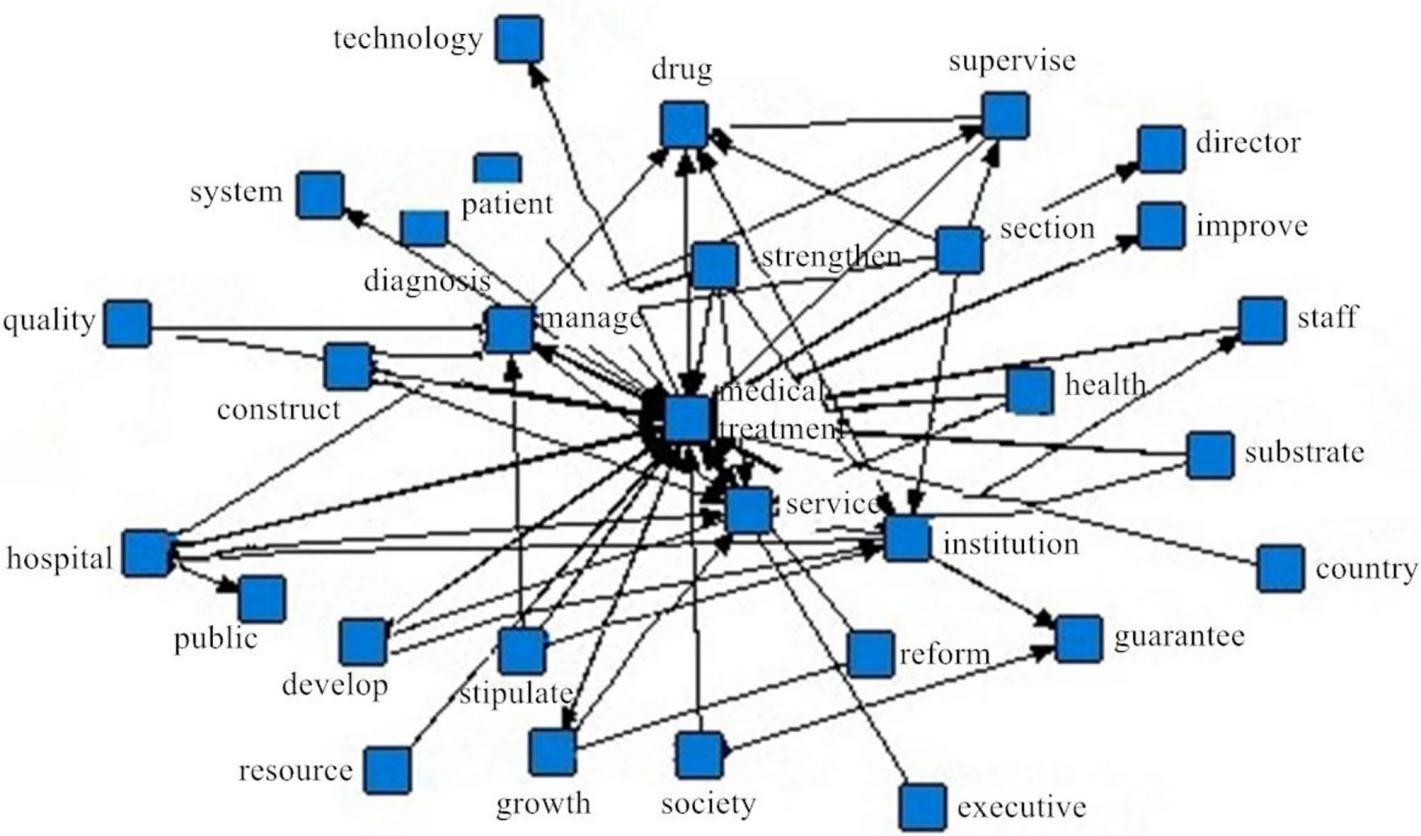
Words network association view of medical service policy text in Sichuan province.

The sample policy documents used in this article were obtained from the official website of the People's Government of Sichuan Province, the Health Committee of Sichuan Province, the Sichuan Provincial Healthcare Security Administration, the Sichuan Drug Administration, and the Sichuan Traditional Chinese Medicine Administration. These government documents were selected according to the following principles: (1) the issuing unit is the Sichuan Provincial Government and its relevant departments; (2) the selected policy document is directly related to the medical policy; and (3) the policy document is issued in the comprehensive medical reform stage. Based on the feasibility of the policy document acquisition, a total of 221 valid samples of policy issued during the period 2010–2018 were collected; some of these are shown in Table 2. By using the ROST software to carry out the word network analysis of the medical service policy text of Sichuan Province in 2010–2018, as shown in Figure 3, the term “medical” was found to have the highest frequency of occurrence (3,104 times). It is also demonstrated that the policy text selected in this paper has a close relationship with the medical treatment.

**Table 2:** Quantitative standards for medical service policy.

**Order** ** number**	**Title**	**Document number**	**Promulgation date**
1	*Circular of the General Office of the Sichuan Provincial people's Government on adjusting the Supervision and Administration system of Food and Drug at or below the Provincial level in Sichuan Province*	No.75 [2010] issued by Sichuan Office	08-27-2010
2	*Circular forwarded by the General Office of the Sichuan Provincial people's Government to the Department of Health of the Provincial Development and Reform Commission and other departments on further encouraging and guiding social capital to organize medical institutions*	No.27 [2011] issued by Sichuan Office	05-31-2011
3	*Circular of the General Office of the Sichuan Provincial people's Government on the issuance of pilot work arrangements for the Reform of Public Hospitals in Sichuan Province in 2011*	No.39 [2011] issued by Sichuan Office	07-27-2011
4	*Opinions on the implementation of the General Office of the Sichuan Provincial people's Government on further strengthening the Construction of the contingent of Rural doctors*	No.47 [2011] issued by Sichuan Office	08-12-2011
5	*Circular forwarded by the General Office of the Sichuan Provincial people's Government to the Department of Health of the Finance Department of the Provincial Development and Reform Commission on the implementation plan for cleaning up and resolving the debts of primary medical and health institutions*	No.56 [2011] issued by Sichuan Office	09-27-2011
221	*Opinions issued by the General Office of the Sichuan Provincial people's Government on carrying out the Construction of “three batches” to promote the High quality Development of traditional Chinese Medicine Industry*	No.98 [2018] issued by The People's Government of Sichuan Province	12-28-2018

### Policy Texts Encoding

The word network association diagram obtained using ROST software reflects the overall situation of medical service policy in Sichuan Province from 2010 to 2018; however, it is impossible to analyze the text content in detail. Therefore, this paper continues to use Nvivo 11 software to further mine and explore the policy text to ensure the integrity of the research results. The main method used is line-by-line coding of the research samples, to determine the research dimensions and keywords according to the relevant data and industry characteristics; this selects “supply-side policy instruments,” “environmental policy instruments,” “demand-side policy instruments,” “policy strength,” and “medical service activities” as tree nodes, constructing sub-nodes under each of these. Nvivo 11 software is used to code the reference points of each node according to the selection of nodes and reference points. If the total amount of text is consistent, the more reference points there are, the more adequate the disclosure of information. The reference points are the reflection of the absolute number of nodes in the policy text.

## Research Content

### Analysis of Policy Instruments for Medical Services

The classification results for policy instruments are obtained from the coding analysis of medical service policy texts. As shown in Table 3, proportion 1 is the proportion of reference points of each sub-policy tool in the policy instruments' category, and proportion 2 is the proportion of each type of policy instrument in the reference points of all sub-policy instruments. This shows that the government has different preferences for the various policy instruments in the development of medical services. Environmental policy instruments (368 items) are the most used in Sichuan provincial government documents, followed by supply-side (317 items) and demand-side policy instruments (58 items). The development of medical services is related to the major livelihood challenges of the country and has unique industry characteristics. The quantity and quality of medical service resources is currently unable to meet the needs of the population. The development of supply-side policy instruments is dependent on the other two policy instruments. In order to solve this supply problem, it is necessary to alleviate the structural imbalance of resource allocation with the help of demand-side policy instruments to further stimulate the development of medical services.

**Table 3:** Statistical results of reference points for policy instruments.

**Tree node**	**Node**	**Number of policy texts**	**Reference points**	**Proportion 1**	**Proportion 2**
Supply-side Policy instruments	Financial support	44	46	14.51%	6.19%
	Information servicec	48	49	15.46%	6.59%
	Talent resources	72	79	24.92%	10.63%
	Science and technology Support	52	65	20.50%	8.75%
	Infrastructure	69	78	24.61%	10.50%
	Subtotal	152	317	–	–
Environmental policy instruments	Preventive measure	39	41	11.14%	5.52%
	Goal programming	92	95	25.82%	12.79%
	Financial service	4	4	1.09%	0.54%
	Regulation and supervision	114	136	36.96%	18.30%
	Strategic measures	34	41	11.14%	5.52%
	Fiscal levy	8	8	2.17%	1.08%
	Guarantee work	40	43	11.68%	5.79%
	Subtotal	187	368	- -	–
Demand-side policy instruments	Government purchase	1	1	1.72%	0.13%
	Market shaping	20	25	43.10%	3.36%
	Overseas communication	16	16	27.59%	2.15%
	Services outsourcing	14	16	27.59%	2.15%
	Subtotal	40	58	–	–

The frequent use of environmental policy instruments reflects the importance attached in Sichuan Province to medical service policy and aims to create a favorable environment for their development through macro policy. Specifically, among the sub-policy environmental policy instruments, regulation and supervision is the most widely used, accounting for 36.96% of reference points. The malpractice in medical service systems and mechanisms in Sichuan Province has not yet been completely removed. In the 13th Five-Year Plan for the Development of Health and Family Planning in Sichuan Province, it is clearly pointed out that the existing costs of medical services is unreasonable, that the reform of a medical insurance payment system is overdue, and the price of drugs and consumables is high. Therefore, the long-term application of a regulatory and supervisory instrument is the highest priority. The second priority is goal programming, accounting for 25.82% of reference points, improving comprehensive medical reform of all aspects of medical security, services, and the drug supply and regulatory system, the implementation of which needs to be carried out in stages, making it necessary to formulate corresponding specific target plans in each stage of reform. The application of strategic and preventive measures and guaranteed work is a high priority, accounting for 11.14, 11.14, and 11.68% of reference points, respectively. The Sichuan Provincial Government has the lowest use of regulation and supervision, and financial services, accounting for 2.17 and 1.09%, respectively. As the fundamental guarantee of the development of medical services, this has not received adequate attention from the government, which needs to encourage the medical development agencies and enterprises to make available innovative financing and investment mechanisms. Furthermore, relevant medical institutions and enterprises should be given assistance in the form of financial subsidies and tax allowances.

Supply-side policy instruments are also used frequently in Sichuan Province. In the sub-policy instruments, the proportion of infrastructure, human resources, and scientific and technological support on the supply-side is 24.61, 24.92, and 20.50% of reference points, respectively. Infrastructure is a basic prerequisite for ensuring the rapid development of the medical service; human resources cannot directly promote the development of the medical industry alone, however, they contribute to improving the utilization and allocation efficiency of other input factors. Scientific and technological support directly promotes the improvement in quality of medical services, and under the remit of the “talent plan” and the current situation, the government should establish a long-term and comprehensive human resource development plan, with specific implementation measures to achieve the strategic objective of developing the provincial medical service. This will accelerate scientific and technological innovation to construct the medical science and technology innovation platform, promote medical scientific research, and to transform and promote medical scientific and technological achievements. Information services account for 15.46% of the total reference points, and in order to effectively promote the development of the three medical institutions, the Sichuan Provincial Government needs to establish a medical service information platform for the province, city, and county, that consists of information services; a management information system of all medical and health institutions; and make full use of available online knowledge and big data to promote the development of medical information. Financial support is 14.51% of reference points; this sub-policy tool has the lowest utilization rate of supply-side policy instruments, and the government should provide more financial support to strengthen the capacity of medical services such as medical institutions (51).

In comparison with the previous two policy instruments, the use of demand-side policy instruments in Sichuan Province is clearly insufficient, with the figures for market shaping at 43.1% of reference points, 27.59% for overseas communication and service outsourcing, and 1.72% for government purchasing. Access to medical treatment is related to people's livelihoods and there will always be a market for this; however, with the improvement of people's living standards, the demand for health care has become more urgent. Therefore, it is necessary to continuously explore ways to extend the health market and to promote the integration of medical services, services for the elderly, and web-based health care services (52). Further, in order to improve access to comprehensive medical services in Sichuan, the government needs to further support the exchange of international medical services in terms of personnel training, information sharing, and scientific and technological exchange.

### Analysis of Policy Strength for Medical Services

**Figure 4:**
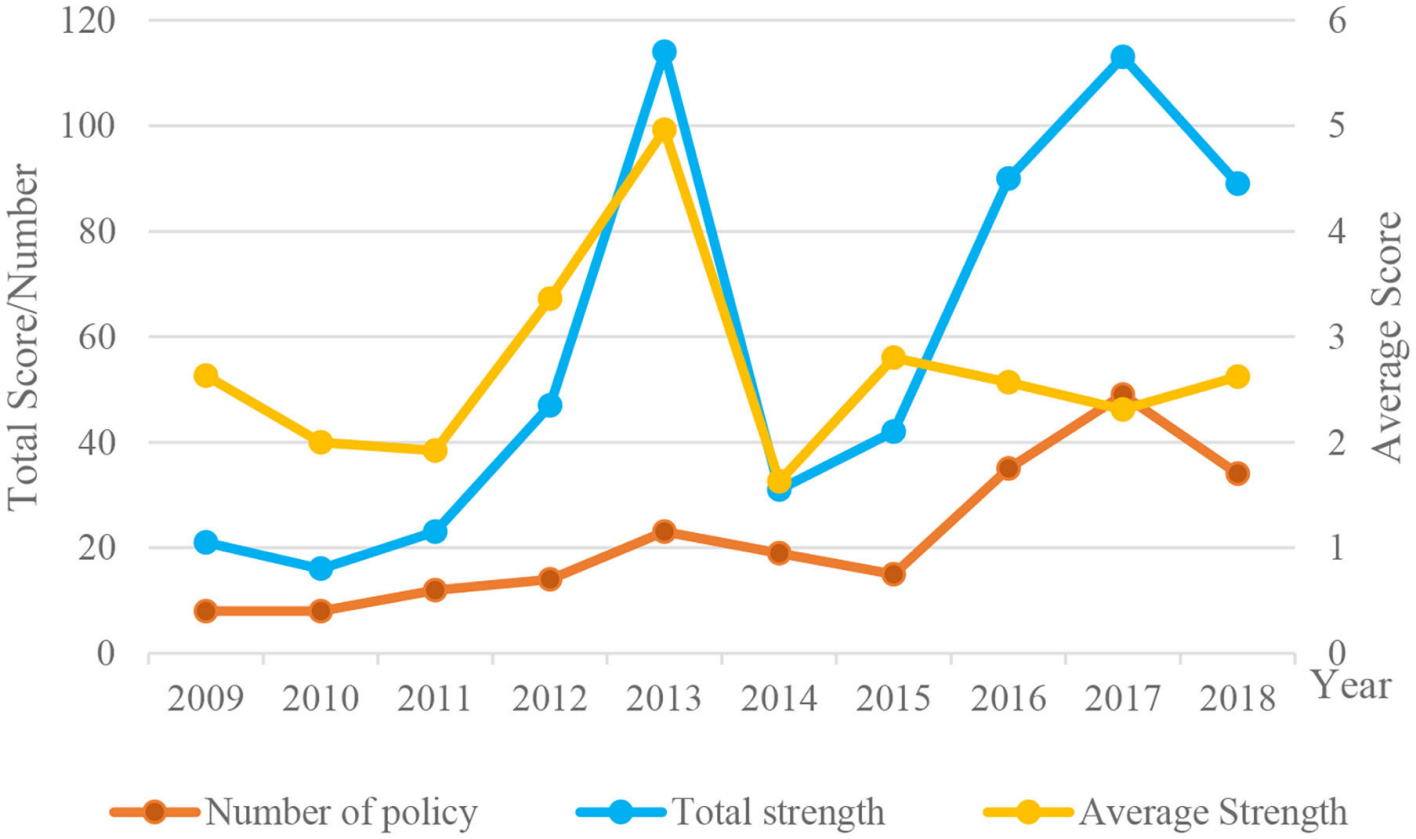
The annual status of the medical service policy release in Sichuan Province.

According to the “1~6” assignment method, the annual status of the medical service policy release in Sichuan Province was obtained (as shown in Figure 4). Viewed from the perspective of the overall policy intensity, 2013 and 2017 represent two peaks. Because the medical service industry is managed at the national level, services in Sichuan Province need to comply with relevant national laws and regulations. In order to resolve the key difficulties and long-term health problems faced by deepening medical service reform, China has focused on strengthening medical and health policy research and establishing an appropriate legal system. In 2013, 17 legislative acts and regulations related to the medical industry were issued, which raised the overall effectiveness of medical policy to its highest level to date. In 2016, the Central Committee of the Communist Party of China and the State Council issued the “The Healthy China 2030 Planning Outline” and the provincial government of Sichuan Provincial Party Committee fully implemented the “Healthy China” strategy and promoted the “Healthy Sichuan” initiative, integrating the promotion of medical security, medical services, and the supply of medicinal drugs. In order to dismantle the obstacles against institutional mechanisms for developing the medical and health care, Sichuan Province vigorously promoted corresponding reform measures and issued a number of policy documents in 2017, including 19 interim regulations, implementation plans, decisions, opinions, methods, and standards; these were out of the total of 24 notices and announcements issued in that year by the Sichuan Provincial People's Government. As a result, the overall strength of policy reached a new peak.

It can be seen from Figure 4 that the average strength of the policies promulgated by Sichuan Province from 2014 to 2018 shows a steady trend, although the number of policies and the overall policy strength show an upward trend particularly in the 3 years from 2014 to 2017. The statistical data show that the number of policy documents promulgated during this period increased year by year, with a consequent increase in overall policy strength. After 2017, the number of policies and corresponding strength of the overall policy show a downward trend. From the statistical data, it is found that the temporary regulations, implementation plans, opinions, and other government documents promulgated in 2018 and 2019, in combination with the circulars and announcements issued by the Sichuan Provincial Government and its committees, bureau and offices, all show a decreasing trend. The development of medical services in Sichuan Province is still facing many problems that need to be solved, therefore it is necessary to strengthen further policies to improve the medical security system, the cost of medical services, and to distribute medical resources fairly in urban and rural areas.

### Analysis of Medical Service Activities

By coding the three categories of medical service activities (medicines, medical, and medical insurance), this paper shows the amount of policy publications related to these different activities in Sichuan Province. Some medical policy documents involve more than one medical service activity. The results of the coding analysis show that there are 84 policy texts related to medicine-related activities, 166 related to medical activities, and 27 to medical insurance activities. The volume of publications for different types of policies shows that medical service activities accounts for the largest proportion of medical activities in Sichuan Province. Medical care has always been an important guarantee of the health of all people in a region. Also, the province attaches great importance to the development of the pharmaceutical industry or production and supply of medicines. In addition to the drug procurement mechanism and innovative drug research and development, the highlight in the field of medicine in Sichuan Province is the development of traditional Chinese medicine. The Province plans to include the traditional Chinese medicine health service industry in the important strategic arrangements for its “5+1” trillion-level modern industrial system.

In this paper, the difference between the policy instruments used in different medical service activities is analyzed by using the matrix query method of Nvivo 11. Firstly, the situation of the first-level policy instruments is analyzed, and the statistical results of the reference point account ratios are shown in Table 4. It is clear that the policy documents of the three medical service activities favor the use of environmental policy instruments, indicating that the Government is focusing on the development of medical services, with continuous optimization of environmental factors to provide a better environment for medical services. The use frequency of the demand-side policy instruments is the lowest, at <10%. In the current society, with a rich material base and the improvement of living standards, the demand for medical services is becoming increasingly urgent, and the market for these services is strong. Medical services therefore need to keep pace with the needs of the population, to improve the medical service level, expand and optimize new innovations, such as smart medical service models, to serve the needs of the people more fully and efficiently. The frequency of use of medical activity policy texts for environmental policy instruments and supply-side policy instruments is at a considerable level. The policy text of the medical insurance activities has not used the demand-side policy instruments completely, and the development of the medical insurance activity market is stable.

**Table 4:** Two-dimensional analysis of reference points between medical service activities and first-level policy instruments.

**Policy** ** instruments**	**Supply-side** ** Policy instruments**	**Environmental** ** policy instruments**	**Demand-side** ** policy instruments**
Medicine activities	39.36%	54.79%	5.85%
Medical activities	44.63%	46.67%	8.70%
Medical insurance activities	36.36%	63.64%	0

In order to understand more clearly the strengths and weaknesses of policy instruments used by each medical service activity and the differences in their use, this paper provides in-depth analysis of the specific use of the sub-policy instruments in the policy documents of the three types of medical service activities; the statistical results of the proportions of reference points are shown in Table 5.

Table 5Two-dimensional analysis of reference points between medical service activities and secondary policy instruments.

**Table:** 

**Supply-side** ** Policy instruments**	**Financial** ** support**	**Information** ** service**	**Talent** ** resources**	**Science and** ** technology support**	**Infrastructure**
Medicine activities	3.19%	4.26%	11.70%	9.04%	11.17%
Medical activities	6.85%	6.85%	10.93%	8.89%	11.11%
Medical insurance activities	9.09%	18.18%	0	0	9.09%

**Table:** 

**Demand-side** ** policy instruments**	**Government** ** purchase**	**Market** ** shaping**	**Overseas** ** communication**	**Services** ** outsourcing**
Medicine activities	0	2.13%	1.60%	2.13%
Medical activities	0.19%	3.89%	2.41%	2.22%
Medical insurance activities	0	0	0	0

**Table:** 

**Environmental** ** policy instruments**	**Preventive** ** measure**	**Goal** ** programming**	**Financial** ** service**	**Regulation and** ** supervision**	**Strategic** ** measures**	**Fiscal** ** levy**	**Guarantee** ** work**
Medicine activities	1.60%	9.57%	0.53%	26.06%	10.11%	1.06%	5.85%
Medical activities	7.22%	13.52%	0.56%	14.63%	4.07%	1.11%	5.56%
Medical insurance activities	0	13.64%	0	27.27%	0	4.55%	18.18%

A total of 188 secondary policy instruments were used in the policy paper on medicine activities. The frequency of regulation and supervision in environmental policy instruments is the highest, accounting for 26.06% of reference points; the use of secondary policy instruments has a frequency of <5% for financial support and information services in the supply-side policy instruments, preventive measures, financial services, and fiscal levy in the environmental policy instruments; and for all demand-side policy instruments, the government purchase of policy instruments has been at zero. A total of 540 secondary policy instruments were used in the policy paper on medical activities, with the highest frequency of regulation and supervision use in environmental policy instruments, accounting for 14.63% of reference points, and the use of secondary policy instruments with a frequency of <5% being financial services, strategic measures, and fiscal levy in environmental policy instruments, as well as all demand-side policy instruments; a total of 22 secondary policy instruments for policy documents on medical insurance activities, with the highest frequency for regulation and supervision in environmental policy instruments, accounting for 27.27%. The use of secondary policy instruments with a frequency of <5% has human resources, science and technology support in the supply-side policy instruments, preventive measures, financial services, strategic measures, and fiscal levy in environmental policy instruments, as well as all demand-side policy instruments; of these, only the utilization of a fiscal levy was not zero and the use of other secondary policy instruments with a utilization rate of <5% was zero.

The above data show that in the use of secondary policy instruments, the structure of policy instruments for each medical activity is different; the difference is most obvious between medical insurance activities and the others. No demand-side policy instruments are used in the policy text of medical insurance activities, and the use of secondary policy instruments in environmental policy instruments and supply-side policy instruments is also very different. The particularity of medical insurance activities does not involve developmental policy instruments such as human resources, scientific and technological support, financial services and strategic measures, and policy instruments such as information services, goal programming, and guarantee work are preferred. However, the most frequently used secondary policy instruments in the policy documents of medicine activities, medical activities, and medical insurance activities are regulation and supervision, indicating their importance in the development of medical services.

## Conclusions

Through the application of content analysis and text mining and using Sichuan Province as an example, medical service policy documents published since the implementation of the new medical reforms are selected and frequency analysis of various policy instruments, policy strength, and medical service activities are applied to draw the following conclusions: The overall composition of policy instruments is uneven, with a high proportion of environmental and supply-side policy instruments, and a serious shortage of demand-side policy instruments. This demonstrates the government's attempts to optimize the environment of medical services, to vigorously improve the supply of related elements of medical development, and to promote the long-term development of medical services: however, the level of attention received by the medical service market is low. The distribution of secondary policy instruments is also uneven. Among the various environmental policy instruments, the Sichuan Provincial Government is more likely to ensure the stable and controllable development of medical services through strong regulation and supervision by the government; this emphasizes the importance of the development of medical services in accordance with the rules and regulations, which is conducive to the improvement of medical service capacity and the protection of the people's basic rights and interests relating to health. However, the proportion of financial services and fiscal levy is very low, which, to some extent, will affect the implementation of target planning in policy documents. In the supply-side policy instruments, there is no significant disparity in the use of secondary policy instruments (human resources and infrastructure). In the demand-side policy instruments, foreign exchange, and service outsourcing policy instruments are low, with the shortage of government purchases warranting the most concern. As can be seen in the structural distribution of policy instruments, the government has focused on regulation, supervision, and goal programming; however, the supporting factors of financial services, in terms of fiscal levies, government purchases, and overseas communication are weak, which limits the pace of development of the medical service market.

In terms of policy strength, 42% of the policy documents are relevant circulars and announcements issued by the Sichuan Provincial People's Government and its committees, bureaus, and offices, while 38% of the policy documents are temporary regulations, implementation plans, decisions, opinions, methods, and standards. The government's policy efforts mainly focus on the goals, operational principles, and clear tasks related to medical services. Secondly, the medical industry mainly abides by the relevant laws and regulations formulated by the state, and the local laws and regulations formulated by the government are secondary. From 2009 to 2018, the overall policy strength of Sichuan Province has changed substantially, but it has been stable in 2016–2018 the past 3 years, and the number of policy issues has also shown an increasing trend, indicating that the government attaches growing importance to the development of the medical industry.

In terms of the types of medical service activities, there are great differences in the number of policy issues between medicine, medical, and medical insurance activities: 60% of the medical service policies are related to medical activities; in contrast, the number of policy documents issued in relation to medical insurance activities is the lowest, at 10%. The policy texts of the three kinds of medical service activities prefer the use of environmental policy instruments, and the frequency in the use of demand-side policy instruments is the lowest, which indicates that the government attaches great importance to the environment of these three medical service activities and constantly optimizes the environmental factors in order to provide a better service environment. The utilization rate of secondary policy instruments in each medical service activity is very different, especially when comparing medical insurance activities with the former two. No demand-side policy instrument is used in medical insurance activities, and the use of secondary policy instruments in environmental and supply-side policy instruments is also very different. There is a serious lack of policy instruments, such as human resources, scientific and technological support, preventive measures, financial services, and strategic measures.

The improvement of the national level of health is essential for the improvement of national productivity, sustainable economic development, and social harmony. The government undertakes the role of the basic demand guarantor, the system supplier, and the market regulator in the medical service through the formulation and implementation of medical service policy in order to safeguard national health. Using the analysis of medical service policy in Sichuan Province as an example, this paper offers policy suggestions on the development of medical service from the following three angles.

From the point of view of policy instruments, the government needs to optimize the their overall structure and the structure of internal secondary policy instruments in order to appropriately reduce the use of environmental policy instruments, increase the application of demand-side policy instruments, and enhance the interaction between secondary policy instruments. In particular, this increases the frequency of use of supportive policy instruments such as government purchases, financial services and fiscal levies, as well as market shaping, overseas communication, and strategic measures, and encourages medical development institutions and enterprises to carry out innovative financing through investment and other measures, while providing support to relevant medical institutions and enterprises in terms of financial subsidies and tax incentives. The government needs to consider the coordinated use of various policy instruments, enhance continuity and stickiness among secondary policy instruments, and focus on strengthening policy synergies in order to enhance the policy effect and improve the sustainability of health care policies.

From the point of view of policy strength, the government must continue to strengthen the policy and accelerate the establishment of basic medical service systems for urban and rural residents. To improve the attitudes toward implementation and make these more conducive to the development of medical services, government policy should be implemented as soon as possible to enable universal access to essential medical services.

From the point of view of medical service activities, the linked development of three kinds of medical service activities should be accelerated. Medicine activities need to focus on improving drug supervision mechanisms, drug supply shortage guarantee mechanisms, and drug purchase mechanisms. Medical activities must aim to strengthen overseas communication and meet the requirements of financial services and fiscal levies, directly provide more support to the service capacity of medical service subjects such as medical institutions in relation to funding, promote the formation of new medical service models such as intelligent medical services, and improve their overall standard. There are still some problems in medical insurance activities, such as weak supervision of medical insurance funds and misappropriation of insurance. It is necessary to strengthen supervision and preventative measures, continue to implement reform of the system, improve the payment mechanism for medical insurance, solve the problems of accessing medical care in various locations, and mitigate commercial risks as soon as possible in order to improve the level of basic medical insurance.

## Data Availability Statement

All datasets generated for this study are included in the article/supplementary material.

## Author Contributions

HZ: project administration, supervision, and writing—review & editing. LZ: data curation, methodology, software, and writing—original draft. CZ: data curation and methodology. XC: methodology, supervision and writing—review & editing. All authors contributed to the article and approved the submitted version.

## Conflict of Interest

The authors declare that the research was conducted in the absence of any commercial or financial relationships that could be construed as a potential conflict of interest.
